# Comparison of Lamotrigine and Oxcarbazepine Monotherapy Among Chinese Adult Patients With Newly-Diagnosed Focal-Onset Epilepsy: A Prospective Observational Study

**DOI:** 10.3389/fneur.2022.855498

**Published:** 2022-06-10

**Authors:** Yuncan Chen, Qinyue Wang, Ye Xu, Dongyan Wu, Lan Xu, Guoxing Zhu, Xunyi Wu

**Affiliations:** ^1^Department of Neurology, Huashan Hospital, Shanghai Medical College, Fudan University, Shanghai, China; ^2^National Center for Neurological Disorders, Shanghai, China

**Keywords:** efficacy, lamotrigine, oxcarbazepine, mood, quality of life, monotherapy

## Abstract

**Objective:**

We performed a prospective cohort study to compare the efficacy, safety, effect on mood, and quality of life between lamotrigine (LTG) and oxcarbazepine (OXC) monotherapy among Chinese adult patients with newly-diagnosed focal-onset epilepsy (FOE) with or without secondarily generalized tonic-clonic seizures.

**Methods:**

We enrolled 106 adult patients with new-onset FOE, of whom 56 were in the OXC group and 50 in the LTG group. Their clinical characteristics were detailly recorded especially basic seizure frequency, seizure types, and drug-related adverse events. Efficacy was evaluated as seizure-free (no seizure for 6 months), effective (seizure reduction by more than 50%), and ineffective (seizure reduction by less than 50%). Both intention-to-treat and per-protocol analyses were performed. We also assessed their mood state with the Zung Self-rating Scale for anxiety (Z-SAS) and Zung Self-rating Scale for Depression (Z-SDS) and quality of life (QOL) with Quality of Life in Epilepsy (QOLIE-31) at their baseline visit, 3-month visits, and 6-month visit. Intra-group comparisons in each group and inter-group comparisons between the two groups were made. Correlation analysis and multiple regression analysis were also conducted.

**Results:**

Except for gender, the two groups were well matched in any other characteristics such as primary seizure frequency and seizure types. In terms of efficacy, 33 patients in the OXC group were evaluated as seizure-free and 15 as effective, while in the LTG group, 31 were seizure-free, and nine were effective. No significant differences could be observed in efficacy between the two groups (*P* = 0.429). Through multiple logistic regression analysis, we found that OXC monotherapy was more likely to predict a seizure-free state (OR = 1.76) than LTG, but the difference didn't reach statistical significance (*P* = 0.322) after correcting for other clinical variables. Both groups had adverse events such as fatigue, drowsiness, dizziness, rash, and gastrointestinal discomfort, most of which were mild and transient. In the OXC group, the scores of SAS (*P* = 0.067) and SDS (*P* = 0.004) reduced at the 6-month visit, while the score of QOLIE-31 significantly increased (*P* = 0.001). In the LTG group, a significant decrease in SAS and SDS scores and an increase in QOLIE-31 scores could be witnessed (All *P* < 0.001). The inter-group comparison showed that improvement of SAS and SDS in the LTG group was more evident than that in the OXC group, which was of statistical significance. Correlational analysis indicated that the improvement of mood and life quality scales in both groups was independent of baseline seizure frequency and treatment efficacy. Multiple linear regression analysis indicated that LTG monotherapy was the only independent factor that could predict a better SAS (*P* = 0.01) and SDS (*P* = 0.019) outcome.

**Conclusions:**

OXC and LTG are effective as monotherapy and can be considered first-line selection among adult patients with new-onset FOE. Most adverse events are mild, transient, and tolerable. The two drugs improve the mood state of patients, though LTG is superior to OXC in this respect. OXC and LTG have great power in enhancing patients' quality of life. The positive effect on the psychosocial well-being of epilepsy patients may be one of the intrinsic pharmacological properties of LTG and OXC.

## Introduction

Epilepsy is one of the most common chronic disabling neurologic disorders, affecting over 70 million people worldwide (1). It brings substantial physical, psychological, social, and economic burdens to individuals, families, communities, and countries, accompanied by unfortunately common misunderstanding, fear, and stigma (2).

Focal-onset epilepsy (FOE) is the most common type among the adult population, according to the latest research (3), which may account for 60% of all epilepsies. For a long time, carbamazepine (CBZ) has been regarded as first-line and standard therapy for patients with focal-onset epilepsy; however, with the marketing of newer anti-seizure medications (ASMs), its use has declined because of its inferior tolerability, enzyme induction, and pharmacokinetic interactions (4). In this case, oxcarbazepine (OXC), the keta-analog of CBZ, becomes a succeeding priority among patients with FOE for clinicians, so does lamotrigine, another probable sodium channel blocker. Previous RCTs show LTG is better than CBZ for drug retention, taking both efficacy and tolerability into account (5). Among second-generation ASMs, OXC and LTG have similar mechanisms to prevent epileptic firing, and guidelines of the American Academy of Neurology (AAN) and American Epilepsy Society (AES) recommend OXC and LTG as a first treatment option for patients with FOE (6). However, few studies comparing these two kinds of monotherapy have been conducted among Chinese adult patients. Therefore, a prominent part of our study is comparing the efficacy and safety of OXC and LTG among Chinese adult patients with FOE.

Patients with epilepsy not only worry about a seizure attack, but they suffer from comorbidities that potentially reduce their life quality and even increase their risk of suicidality (7). Psychiatric comorbidities are common among patients with epilepsy (PWE), especially depression and anxiety. Though the mechanism behind it has not been fully elucidated so far, existing evidence from scarce studies implies a bidirectional relation between epilepsy and psychiatric comorbidities (8). Thus, in addition to focusing on reaching a seizure-free state, it's essential to factor in the existence of comorbidities to gain a comprehensive therapy.

Based on such a background, it's necessary and clinically significant to explore the psychotropic properties of ASMs. The positive psychotropic property of LTG has been well known. Studies examining the effects of LTG on mood and quality of life (QOL) have been conducted, which brought plenty of high-quality evidence to identify it as a mood stabilizer (9, 10). However, this kind of effect of OXC, another probable blocker of the voltage-gated sodium channel, is disputable because some studies show a positive impact on the mood of OXC (11). In contrast, others reveal that OXC was one of the independent variables associated with depression (12). Therefore, more reliable studies are needed to investigate the psychotropic property of OXC. Additionally, to our knowledge, there is no available study that compares the effect on mood and QOL between LTG and OXC monotherapy.

Therefore, in the present study, we aim to compare the efficacy, safety, effect on mood and QOL between LTG and OXC monotherapy in Chinese adult patients with newly diagnosed focal-onset epilepsy, with or without secondarily generalized tonic-clonic seizures.

## Methods

### Subjects

Patients with focal-onset epilepsy who visited Huashan Hospital of Fudan University from June 2020 to June 2021 were enrolled in this study. Participants meet the following criteria: age between 14 and 65 years old; newly diagnosed focal-onset epilepsy (secondary to generalized tonic colonic seizure or not) based on clinical presentations and EEG confirmation, according to International League Against Epilepsy classifications of seizures and epilepsy; monotherapy with OXC or LTG according to clinicians' preference or patients' option; capable of completing our questionnaires. Patients taking anti-depressant or other psychotropic medications, or with known or diagnosed psychiatric disorders, or with severe renal or liver diseases are excluded. The study was reviewed and approved by the Medical Ethics Committee of Huashan Hospital of Fudan University. Informed consent was obtained from all participants.

To calculate the sample size, we used a formula which was used to compare two proportions (13) (two-sample and two-sided equality). The α and β value were set at 0.05 and 0.2, respectively. According to previous studies and expert's experience, the seizure-free rate of OXC monotherapy among FOE patients was estimated between 60%−80% (14, 15) while for LTG, 60%−70% (16).

### Evaluation of Outcome

During the study, we assessed our participants and made detailed records about seizure frequency, adverse events, scores of moods, and quality of life at the baseline visit, 3-month visit, and 6-month visit. We chose Zung Self-rating Scale for anxiety (Z-SAS), Zung Self-rating Scale for Depression (Z-SDS), and quality of life in epilepsy (QOLIE-31) for mood state and life quality evaluation. In our study, seizure frequency was evaluated on a four-point scale (17): yearly (1–11 seizures a year), monthly (1–4 seizures a month), weekly (1–6 seizures a week), or daily (>6 seizures a week). Basic characteristics such as gender, age, weight, seizure onset, course of the disease, seizure types, magnetic resonance imaging (MRI) results, electroencephalography, family seizure history, and febrile seizure history were recorded at the enrollment. Additionally, those who dropped out were marked, and their reasons were documented for both intention-to-treat (ITT) and per-protocol (PP) analysis.

We had two groups: the LTG group and the OXC group. Changes in seizure frequency, mood score, and quality of life were compared in each group during the subsequent visit to investigate each drug's anti-epileptic and psychotropic properties. A comparison between groups was also performed.

The effectiveness of the treatment at the last follow-up was evaluated according to the four-grade therapeutic effect criteria developed by the 1st National Epilepsy Academic Conference of the Chinese Medical Association (18) and we made some modulation (grade 2 and 3 merge to be “effective”). The method of evaluation was also used by many previous studies (18–20). Efficacy was categorized as seizure-free (no seizure during the observation period), effective (seizure frequency reduced by >50%), and ineffective (seizure frequency reduced by <50%).

A quantitative assessment of our participants' mood states was performed using Z-SAS and Z-SDS. Z-SAS and Z-SDS are self-administered scales for assessing anxiety and depression among adults. With 20 items, the two questionnaires have a maximum score of 80, respectively, while the higher score represents the more severe state of depression and anxiety. The cut-off score of Z-SAS and Z-SDS is 40.

The QOLIE-31 (21) is an epilepsy-specific, 31-item instrument developed for rapid evaluation of health-related QoL including seven domains: overall QoL (QOL), seizure worry (SW), emotional well-being (EWB), energy/fatigue (EF), cognitive functioning (CF), medication effects (ME), and social functioning (SF). Higher scores reflect a better QoL.

### Statistical Analysis

Statistical analyses were performed using SPSS. Continuous variables were reported as means ± standard deviation (SD) while categorical variables were reported as count and percentage. For comparison of efficacy, both ITT and PP analyses were performed. When evaluating mood and quality of life, PP analysis was used based on available data. Multiple regression analysis was performed to prevent some potential bias. The Chi-square test is used to examine the between-group difference in terms of seizure types, primary frequency, and efficacy. As for the intra-group comparison of mood and life quality scales of each point of time, Paired t-test was used for analysis while a student's unpaired t-test was used to compare relevant indicators between two groups. The Pearson test is used during correlation analysis if we have a dichotomous or ranked variable and a continuous variable; otherwise, the Spearman test is chosen. The significance level was set at the 95% confidence limit. A two-tailed *P* < 0.05 was considered statistically significant.

## Results

### Demographics

**Figure 1:**
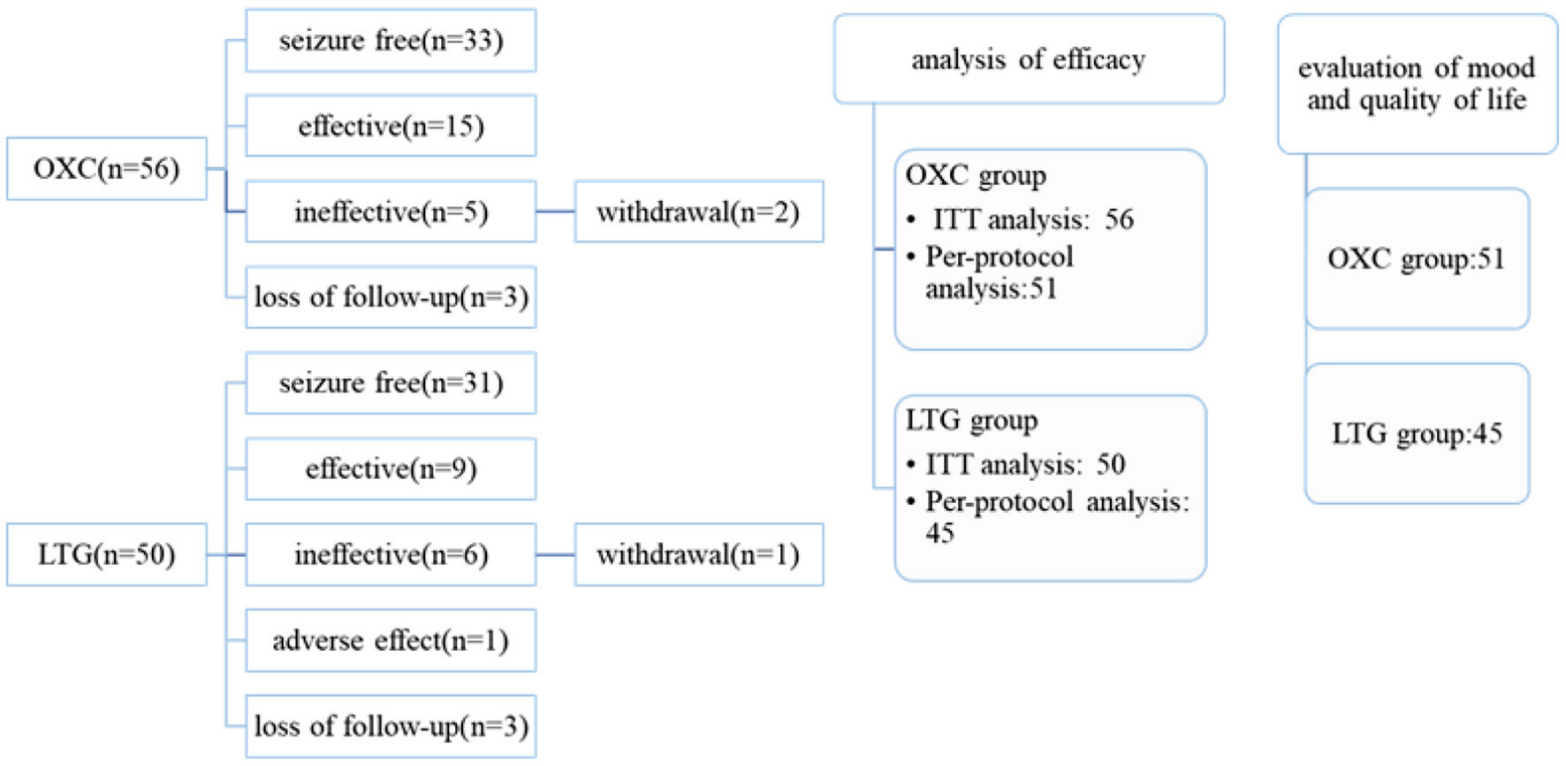
Schematic diagram for patients receiving LTG and OXC monotherapy.

Fifty LTG-treated and 56 OXC-treated patients were enrolled at screen visits; however, 45 in the LTG group and 51 in the OXC group completed the study and were included in the final analysis (Figure 1). There is a significant difference in the male-to-female ratio (*P* = 0.001) and mean weight (*P* = 0.003) between the two groups. Other clinical factors such as age, age of seizure onset, type of seizure, and primary seizure frequency are well matched. Demographic and epilepsy-related characteristics of recruited patients at baseline were detailed in Table 1.

**Table 1:** Clinical characteristics of participants receiving lamotrigine or oxcarbazepine monotherapy.

**Group**	**OXC(*N* = 56)**	**LTG(*N* = 50)**	***P*** **-Value**
Male, *n* (%)	35 (62.5)	15 (30)	**0.001**
Female, *n* (%)	21 (37.5)	35 (70)	
Age^*^, years (mean ± SD)	30.04 ± 11.1	26.5 ± 11.2	0.106
Age of onset^*^, years (mean ± SD)	26.05 ± 11.2	22.34 ± 11.1	0.09
Duration of disease^*^, (mean ± SD)	3.95 ± 4.2	4.55 ± 5.7	0.554
Abnormal MRI, *n* (%)	18 (32.1)	9 (18)	0.095
Abnormal EEG, *n* (%)	28 (50)	30 (60)	0.302
**Type**			
FAS, *n* (%)	6 (10.7)	2 (4)	0.423
FIAS, *n* (%)	12 (21.4)	11 (22)	
FBTCS, *n* (%)	38 (67.9)	37 (74)	
**Basic frequency**			
Yearly, *n* (%)	26 (46.4)	25 (50)	0.979
Monthly, *n* (%)	21 (37.5)	17 (34)	
Weekly, *n* (%)	6 (10.7)	5 (10)	
Daily, *n* (%)	3 (5.4)	3 (6)	

*OXC, oxcarbazepine; LTG, lamotrigine; MRI, magnetic resonance imaging; EEG, electroencephalography; FAS, focal aware seizures; FIAS, focal impaired awareness seizures; FBTCS, focal-bilateral tonic clonic seizures*.

* *Mean ± standard deviation. Bold Value indicates the p < 0.05*.

### Efficacy and Tolerability

**Figure 2:**
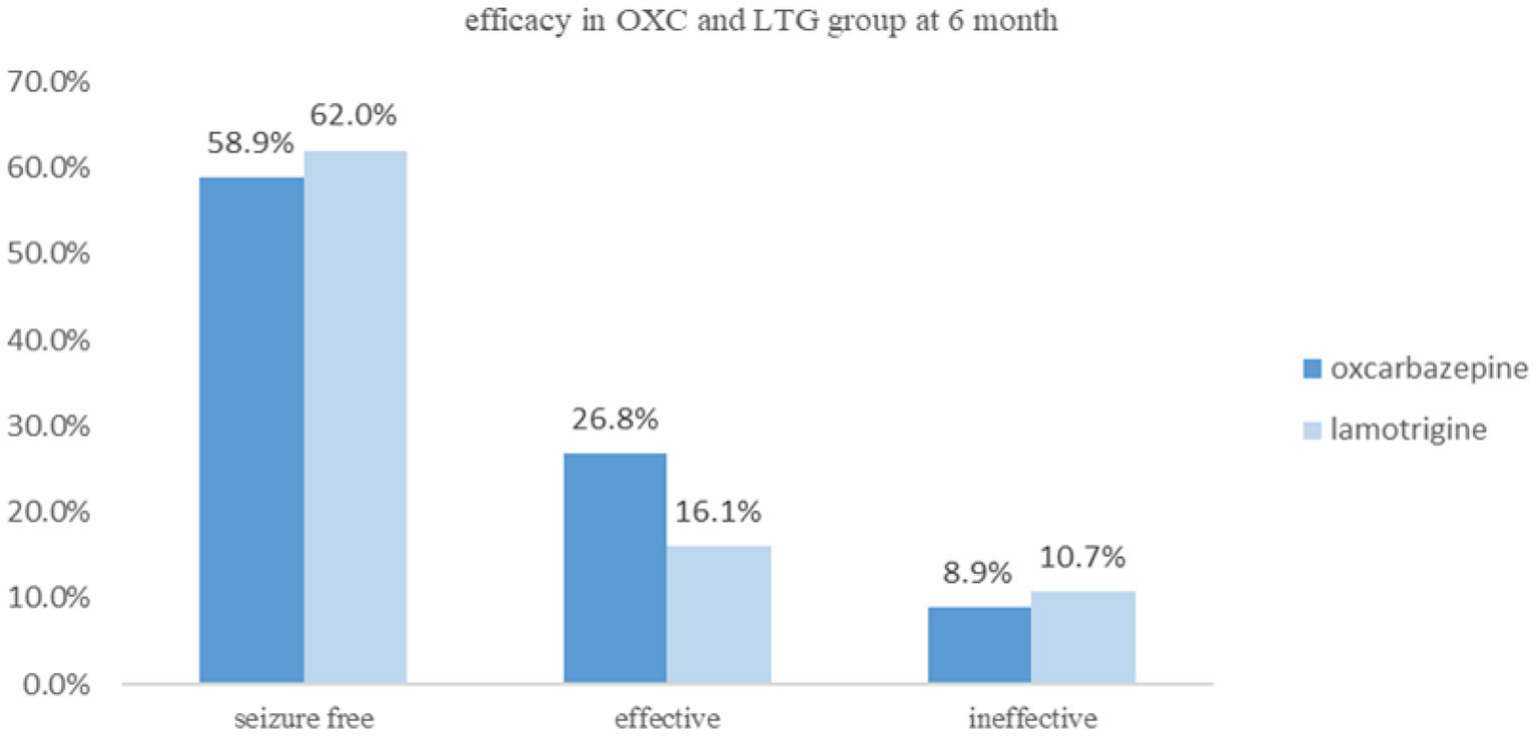
Comparison of efficacy between OXC and LTG at 6 months follow-up.

During the 6-month follow-up, in the OXC group, 33 patients had a seizure-free outcome, and 15 patients had a reduction in seizure by more than 50%. However, six patients who received OXC treatment were evaluated as ineffective, which caused two cases of discontinuation. While in the LTG group, 31 patients were assessed as seizure-free and nine as effective. Six LTG-treated participants were classified as ineffective, and one of them changed to valproate acid. Nevertheless, ITT analysis showed that no significant differences could be observed for efficacy between the two groups (*P* = 0.429, Figure 2). Similar results were obtained in the Per-protocol analysis. Both teams have complaints such as fatigue, drowsiness, dizziness, rash, and gastrointestinal discomfort in side effects. Most side effects were mild, transient, and didn't cause discontinuation, which disappeared as the treatment progressed. Interestingly, we had four patients in the LTG group experiencing a rash, and two of them were asked to start with a lower dose which turned out to be rash-free, while one case had to change to another anti-epileptic drug. Some side effects observed among our cohort are listed in Table 2.

**Table 2:** Treatment-related adverse events.

**Adverse events**	**Oxcarbazepine**	**Lamotrigine**
Fatigue	1	1
Drowsiness	3	1
Dizziness	3	2
Gastrointestinal discomfort	1	2
Rash	1	4
Impaired memory	1	0
Impaired hepatic function	1	0

In multiple logistic regression analysis, we found that OXC monotherapy was more likely to predict a seizure free state (OR = 1.76, Table 3) than LTG, but the difference didn't reach a statistical significance (*P* = 0.322) after correcting for other clinical variables. Additionally, we found basic frequency of “daily” was an independent factor that could predict a seizure free state (OR = 12.86, *P* = 0.014).

**Table 3:** Multiple logistic regression analysis for seizure free outcome.

**Factor**	**β**	**Odd ratio (95% CI)**	***P*** **-Value**
Age	−0.183	0.83 (0.56–1.23)	0.359
Age at seizure onset	0.138	1.15 (0.77–1.70)	0.494
Disease course	0.336	1.40 (0.92–2.12)	0.114
OXC monotherapy	0.563	1.76 (0.58–5.35)	0.322
Male	0.01	1.01 (0.34–3.02)	0.985
FAS^*^	1.247	3.48 (0.57–21.26)	0.177
FIAS^*^	0.746	2.11 (0.55–8.10)	0.277
Frequency (daily)^**^	2.554	12.86 (1.69–97.81)	**0.014**
Frequency (weekly)^**^	1.461	4.31 (0.80–23.13)	0.088
Frequency (monthly)^**^	0.411	1.51 (0.50–4.53)	0.464
MRI abnormality	−1.078	0.34 (0.09–1.30)	0.115
EEG abnormality	0.444	1.56 (0.54–4.48)	0.41

*OXC, oxcarbazepine; MRI, magnetic resonance imaging; EEG, electroencephalography; FAS, focal aware seizures; FIAS, focal impaired awareness seizures*.

* *Compared with focal bilateral tonic clonic seizures*.

** *Compared with baseline frequency of “yearly”*.

*Bold Value indicates the p <0.05*.

### Effect on Mood and Quality of Life

#### Intra-Group Comparison in Each Group

**Figure 3:**
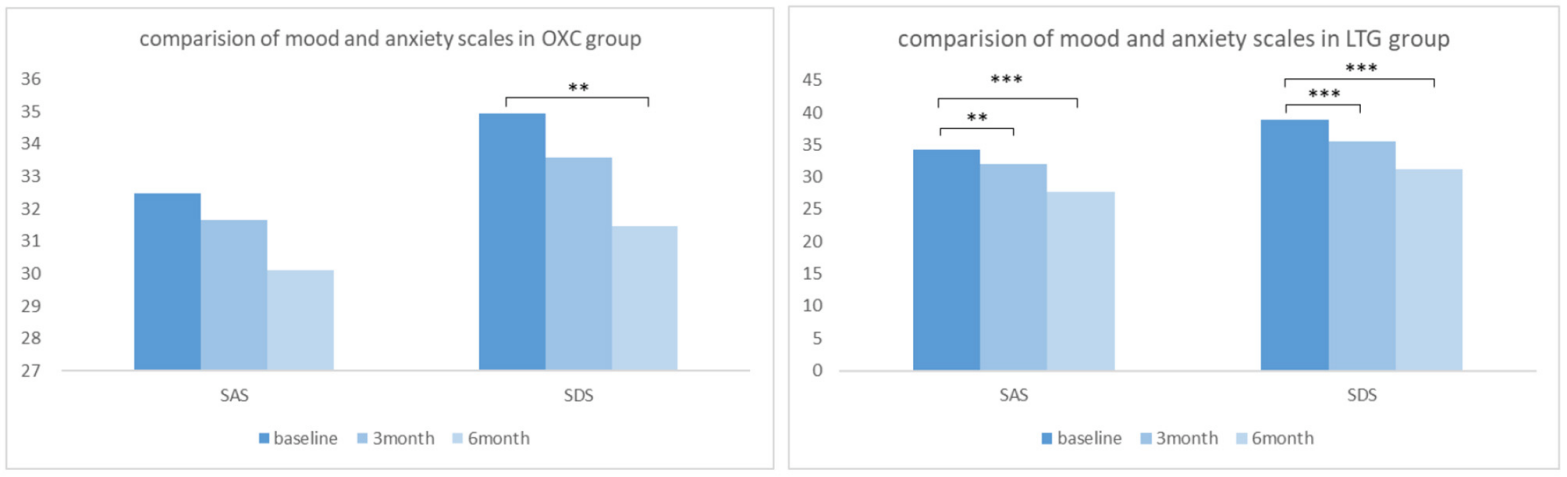
Comparison of mood and anxiety scales in OXC and LTG group respectively. OXC, oxcabazepine; LTG, lamotrigine; SAS, self-rating anxiety scale; SDS, self-rating depression scale. ^**^P < 0.01; ^***^P < 0.001.

In the OXC group, the raw average scores of SAS and SDS of our patients before medication were 32.47 ± 8.49 and 34.94 ± 9.79, respectively. At 3- and 6-month follow-up, reduction of scores in both scales could be witnessed (Figure 3), indicating improvement of mood and anxiety state. Notably, the 6-month score of SDS is significantly better than that of baseline (*P*< 0.05). While in the LTG group, the average scores of SAS and SDS of participants at baseline were 34.29 ± 8.04 and 38.84 ± 8.98, respectively. There was a significant reduction of SAS and SDS scores at both 3- and 6-month follow up (*P*< 0.01).

**Figure 4:**
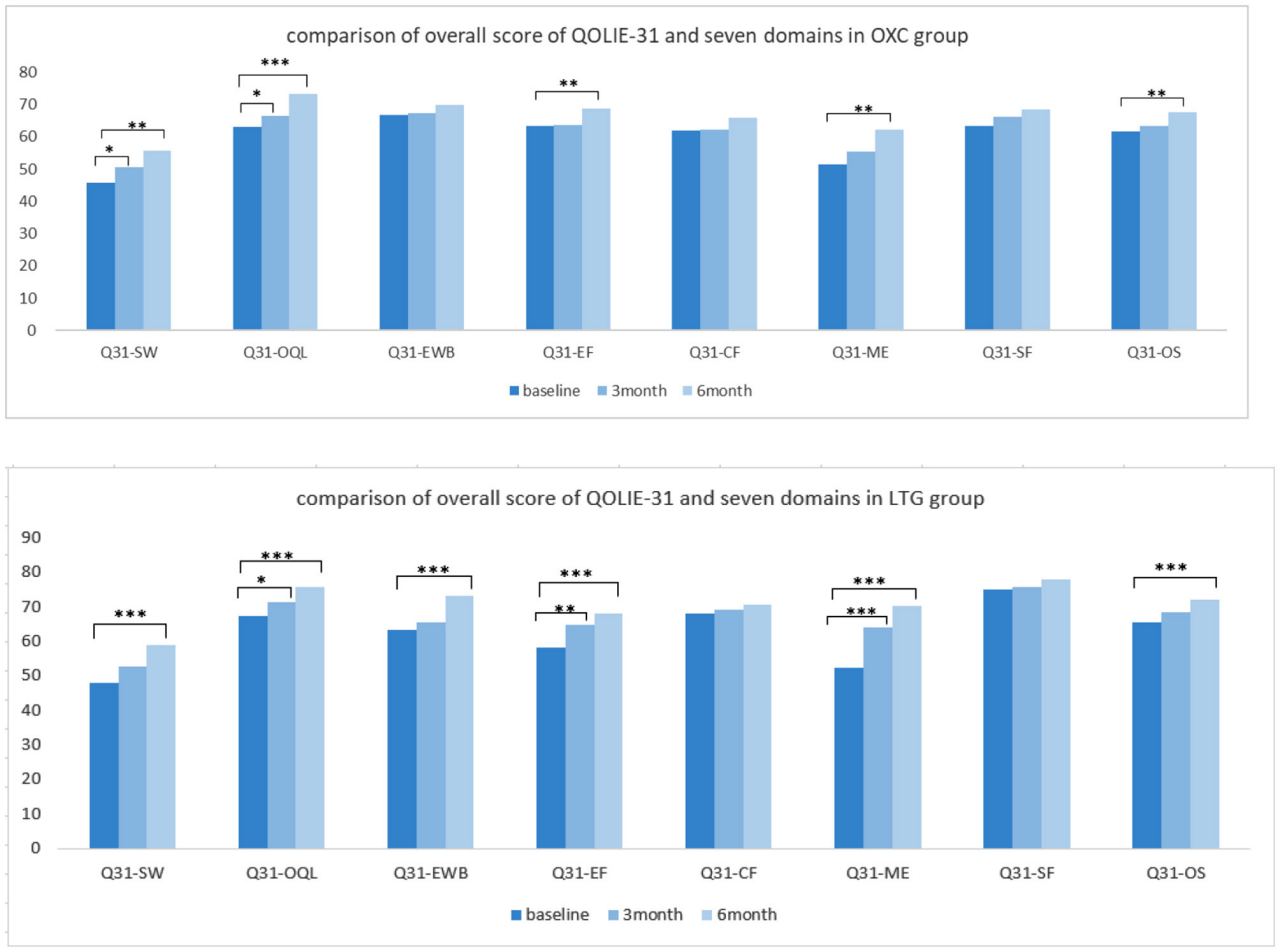
Comparison of quality of life independently in each group. OXC, oxcabazepine; LTG, lamotrigine; Q31, QOLIE-31; SW, seizue worry; QOL, overall quality of life; EWB, emotional wellbeing; EF, energy/fatigue; CF, cognitive functioning; ME, medication effect; SF, social functioning; OS, overall score. ^*^P < 0.05; ^**^P < 0.01; ^***^P < 0.001.

Regarding the quality of life, participants in the OXC group showed a significant improvement in quality of life after a 6-month therapy (*P* < 0.01). More specifically speaking, positive outcomes with significance could be observed in domains such as seizure worry, overall QOL, energy-fatigue, and medication effects. In the LTG group, the overall score of QOLIE-31 got better with statistical significance (*P* < 0.001), so as almost all the domains except cognitive and social functioning (Figure 4).

#### Correlational Analysis in Each Group

To figure out what factors may contribute to the decrease of the score of SAS and SDS and increase of score of QOLIE-31, a correlation analysis was conducted. It indicated that in each group, the patients' gender and age, the baseline seizure frequency, treatment efficacy, and EEG result have weak relation with change of scores of mood scales and QOLIE-31, no matter negative or positive. One exception is that in the LTG group, patients' fundamental frequency is moderately related to the decrease of SDS score. As for mood scales, there is a significantly strong positive correlation between the decline of SAS and SDS. Additionally, the change of SAS and SDS is moderately negatively correlated with the evolution of the score of QOLIE-31 with statistical significance (Table 4).

**Table 4:** Correlation analysis for change of scores of SAS, SDS, and QOLIE-31 in each group.

**Correlation coefficient**	**OXC**			**LTG**		
	**ΔSAS**	**ΔSDS**	**ΔQ31-OS**	**ΔSAS**	**ΔSDS**	**ΔQ31-OS**
Gender	-0.018	-0.048	-0.05	0.093	0.029	0.102
Age	-0.113	-0.221	0.154	-0.125	-0.144	-0.029
Baseline frequency	-0.006	-0.077	0.085	0.221	0.40^**^	-0.102
Effectiveness	0.047	-0.167	0.035	0.123	0.089	-0.157
EEG	-0.126	-0.168	0.09	-0.125	-0.144	-0.029
ΔSAS	-	0.626^**^	-0.582^**^	-	0.637^**^	-0.501^**^
ΔSDS	-	-	-0.57^**^	-	-	-0.266^**^

*OXC, oxcarbazepine; LTG, lamotrigine; EEG, electroencephalography; Δ, scores change at 6-month follow-up compared with baseline; SAS, self-rating anxiety scale; SDS, self-rating depression scale; OS, overall score; Q31, QOLIE-31*.

*Bold Value indicates the p < 0.05*.

#### Inter-group Comparison Between Two Groups

To reveal to what degree the two drugs affect the mood and quality of life of patients with epilepsy and which drug brings more influence. Compared with baseline, at 6-month follow-up, scores of both the SAS and SDS decreased greater, and scores of QOLIE-31 increased greater in the LTG group than those in the OXC group. Additionally, changes of scores of SAS and SDS were more evident in the LTG group, which reached statistical significance (*P* = 0.007 and *P* = 0.01, Table 5). As for the quality of life, participants who received LTG gained more scores after 6-month treatment, though not significant (*P* = 0.766).

**Table 5:** Comparison of mood and life quality scales after 6-month therapy between two groups.

**Scales**	**OXC**			**LTG**			***P*** **-Value**
	**Baseline**	**6-month**	**Δ**	**Baseline**	**6-month**	**Δ**	
SAS	32.47 ± 8.491	30.12 ± 8.97	−2.35 ± 8.98	34.29 ± 8.04	27.69 ± 5.41	−6.6 ± 5.54	**0.007**
SDS	34.94 ± 9.791	31.47 ± 8.55	−3.47 ± 8.09	38.84 ± 8.98	31.24 ± 6.19	−7.6 ± 7.27	**0.01**
Q31-OS	61.67 ± 14.73	67.69 ± 12.94	6.02 ± 11.71	65.34 ± 12.30	72.03 ± 9.36	6.69 ± 9.84	0.766

*OXC, oxcarbazepine; LTG, lamotrigine; Δ, scores change at 6-month follow-up compared with baseline; SAS, self-rating anxiety scale; SDS, self-rating depression scale; OS, overall score; Q31, QOLIE-31*.

*Bold Value indicates the p <0.05*.

Multiple linear regression analysis indicated that LTG monotherapy was the only independent factor that could predict a better SAS (*P* = 0.01) and SDS (*P* = 0.019) outcome (Table 6).

**Table 6:** Multiple linear regression analysis for outcome of mood and quality of life.

**Scale**	**ΔSAS**		**ΔSDS**		**ΔQ31-OS**	
**Factor**	***B***	***P*** **-Value**	***B***	***P*** **-Value**	***B***	***P*** **-Value**
Constant	5.528	0.435	11.992	0.096	−2.331	0.815
LTG monotherapy	−4.435	**0.01**	−4.087	**0.019**	1.161	0.627
Gender	0.271	0.878	−0.585	0.743	−0.317	0.816
Age	−0.021	0.794	−0.098	0.225	0.23	0.042
Course	−0.074	0.693	0.163	0.393	0.119	0.653
Seizure types	0.594	0.684	−0.575	0.697	−0.545	0.791
Baseline frequency	−1.304	0.219	−1.421	0.186	0.567	0.704
Treatment outcome	−0.547	0.704	−1.571	0.283	−0.068	0.973

*LTG, lamotrigine; Δ, scores change at 6-month follow-up compared with baseline; SAS, self-rating anxiety scale; SDS, self-rating depression scale; OS, overall score; Q31, QOLIE-31*.

*Bold Value indicates the p <0.05*.

## Discussion

In this prospective cohort study, a total of 106 Chinese adult patients were enrolled, and 96 participants got into the last analysis, with 10 cases of drop-out. One patient turned to Chinese traditional medicine, one patient stopped taking the prescribed drug because of worrying about the adverse effect, and four patients didn't come back for a recheck and refused to answer our phone call or online follow-up. Additionally, four patients withdrew because of a lack of efficacy or intolerable side effects. We conducted both ITT and PP analysis when evaluating the efficacy and gained similar results, so we thought the drop-outs might not significantly influence our results.

Clinical data is limited about comparing the effectiveness and safety of LTG and OXC as monotherapy among the adult population with FOE. Our study found that LTG was as effective as OXC for patients with focal-onset epilepsy as monotherapy. The two terms were well matched in seizure types (*P* = 0.423) and frequency (*P* = 0.979) at baseline evaluation. At the last follow-up, there were 33 patients entering seizure-free status, 15 gaining 50% or more seizure reduction, and five being judged as ineffective in the OXC group. While in the LTG group, there are 31 cases of seizure-free, nine gaining 50% or more seizure reduction, and six patients reduced seizure frequency by <50%. There is no significant difference between the two terms as far as effectiveness is concerned in our study. In the actual clinical setting, OXC or CBZ is still considered the first choice though LTG is gaining more and more attention, mainly because high-level evidence is lacking to support LTG. The result of our study, to some extent, echoes with another recent Chinese cohort study which turned out that LTG was significantly better than OXC in terms of a three-year seizure-free rate, and the former was more effective in preventing the first seizure (22). Future studies with more strictly-designed methods of less bias, larger samples, and more extended follow-up period are needed to provide more convincing evidence.

What is worth mentioning is that several older patients in the LTG group entered a seizure-free state. LTG is recommended in patients aged over 60 years with new-onset focal epilepsy in the latest guideline of the American Academy of Neurology (AAN) (6), based on some high-level evidence of comparison with CBZ (23, 24). According to the epidemiology of epilepsy, geriatric patients are expanding their segment in the whole epilepsy population. Still, the extrapolation of outcome data from studies among the younger to older patients is limited due to different etiology and altered pharmacodynamic sensitivity (24). Therefore, more unique and controlled studies among the senior population are needed for further exploration.

Likewise, to previous reports, our study indicates that most of the side effects of LTG and OXC are mild and transient. LTG group had a higher frequency of rash. Of all the four cases of rash in the LTG group, two discontinued and switched to another ASMs (anti-seizure medicines), while the other two patients chose to titrate with a lower starting dose and then turned out to be rash-free. There are several studies indicating that the risks of rash induced by LTG are clinically associated with over-rapid titration (25, 26); therefore, a slower LTG titration and strict adherence to clinicians' dosing guidelines are recommended.

In fact, the new proposed definition of epilepsy by ILAE conveys a need to factor in the existence of psychiatric comorbidities. A recent meta-analysis suggested the overall pooled prevalence of anxiety and depressive disorder among patients with epilepsy (PWE) was 20.2 and 22.9%, respectively (27). Comorbid psychiatric conditions negatively implicate PWE at several levels: predicting risk of refractory epilepsy, premature death, poor quality of life (QOL), and reducing tolerance to AEDs (8). In this study thus, a significant part of our work is dynamically assessing the mood state and quality of life of participants to compare the change in each group and between the two groups.

In our study, we found that LTG remarkably improved patients' mood state at the first and second follow-up, while in the OXC group positive change could only be observed at SDS. A similar result was gained in a Korean self-control study which showed no significant difference on anxiety scales after OXC initiation (28). During the inter-group comparison, we found that LTG was significantly better than OXC in improving our patients' anxiety and depression scales which meant OXC was not as good as LTG in terms of alleviating psychotropic comorbidities. Also, multiple linear regression analysis indicated that LTG monotherapy was the only independent factor that could predict a better SAS and SDS outcome. This result is in accordance with our expectations. After all, the role of LTG as a mood stabilizer is supported by several high-level RCTs (9, 10), while OXC is considered to possess mood-stabilizing properties only based on some anecdotal reports.

In addition to mood effect, ameliorating the quality of life is also a popular treatment objective when clinicians tailor AEDs therapy and select the most appropriate compound. In our study, both groups showed a noticeable improvement in quality of life through self-control. Still, the difference between the two groups didn't reach statistical significance, suggesting OXC and LTG have similar power in enhancing the well-being of patients. In the LTG group, improvement can be observed at not only the overall score of QOLIE-31 but also subdomains such as seizure worry (SW) and emotional well-being (EWB), which is in line with the positive effect in mood state. However, in the OXC group, the improvement in domain of emotional well-being (EWB) didn't reach a statistical significance, which was matched with its performance in mood scales. The domain of the cognitive function (CF) shows slight but not significant improvement in both groups. A similar result can be observed in the study of Seo et al., which indicated that OXC and LTG monotherapy had similar, slightly beneficial effects on cognitive function (29).

Our study is, to our knowledge, the first prospective cohort study to compare the effect on mood and quality of life between OXC and LTG. What is worth mentioning is that we've gone deeper and further by performing both intra-group and inter-group comparison, which suggested LTG was superior to OXC in enhancing the emotional well-being of patients with epilepsy. They have similar great power in improving patients' quality of life.

Furthermore, correlational analysis, the last part of our study, demonstrated that the improvement of mood and life quality scales was not associated with treatment effectiveness, which means the positive effect of LTG or OXC on the psychosocial well-being of epilepsy patients is not (at least not wholly) secondary to seizure reduction. Similar outcomes were also obtained in other studies (9, 10).

When prescribing OXC, one of the major adverse events clinicians worry about is hyponatremia. There is a study showing age over 40 years is one of the main risk factors for hyponatremia among patients taking OXC or CBZ (30). Additionally, female patients are at higher risk for hyponatremia than male patients. Maybe that's why we have more older and female patients in the LTG group; after all, the enrolled patients were placed into LTG or OXC monotherapy mainly according to the preference of the experts.

Our study presents some limitations. Our sample size is not big enough, and it's not a randomized, double-blind, placebo-controlled study that is still deemed “gold standard,” providing the highest-level clinical evidence. The participants included were arbitrarily placed into LTG or OXC group according to the preference of the experts, which may bring selection bias.

Nevertheless, there are some commendable points in this study. We endeavor to keep in contact with all participants to keep the rate of drop-out as low as 9.4% (10/106), and only six patients became lost to follow up. More importantly, it's not cross-sectional, but a prospective study conducted with intra-group and inter-group comparisons.

## Conclusion

Both OXC and LTG are effective as monotherapy and can be considered as first-line selection among eastern Chinese adult patients with new-onset FOE. Most adverse events are mild, transient, and tolerable. The two drugs improve the mood state of patients, though LTG is superior to OXC in this respect. OXC and LTG both have great power in enhancing patients' quality of life. Seizure reduction cannot fully account for mood and life quality improvement, which means they have some potential favorable emotional properties independent of anti-epileptic activity.

## Data Availability Statement

The original contributions presented in the study are included in the article/supplementary material, further inquiries can be directed to the corresponding author.

## Ethics Statement

The studies involving human participants were reviewed and approved by the Medical Ethics Committee of Huashan Hospital of Fudan University. Written informed consent to participate in this study was provided by the participants' legal guardian/next of kin.

## Author Contributions

YC wrote the manuscript. XW and GZ contributed to the conception of the study. QW helped perform the statistical analysis. DW and LX helped collect the clinical data. YC and YX performed the follow-up. All authors contributed to the article and approved the submitted version.

## Funding

This work was supported by grants from the National Natural Science Foundation of China (No. U1909209), Shanghai Municipal Science and Technology Major Project (No. 2018SHZDZX01), and ZJ Lab.

## Conflict of Interest

The authors declare that the research was conducted in the absence of any commercial or financial relationships that could be construed as a potential conflict of interest.

## Publisher's Note

All claims expressed in this article are solely those of the authors and do not necessarily represent those of their affiliated organizations, or those of the publisher, the editors and the reviewers. Any product that may be evaluated in this article, or claim that may be made by its manufacturer, is not guaranteed or endorsed by the publisher.
